# Interstitial lung abnormalities: a mechanistic window into early fibrotic lung disease

**DOI:** 10.3389/fmed.2026.1867568

**Published:** 2026-09-07

**Authors:** Brintha Selvarajah, Durdica Buconjic, Richard J. Hewitt

**Affiliations:** 1 Centre for Inflammation and Tissue Repair, UCL Respiratory, Rayne Institute, University College London, London, United Kingdom; 2 Tumour Cell Biology Lab, The Francis Crick Institute, London, United Kingdom; 3 Interstitial Lung Disease, Respiratory Medicine, University College London Hospitals NHS Foundation Trust, London, United Kingdom; 4 Department of Chemical Sciences, University of Limerick, Limerick, Ireland; 5 King’s Centre for Lung Health, Peter Gorer Department of Immunobiology, School of Immunology and Microbial Sciences, King’s College London, London, United Kingdom; 6 Interstitial Lung Disease Unit, Royal Brompton Hospital, Guy’s and St Thomas’ NHS Foundation Trust, London, United Kingdom

**Keywords:** idiopathic pulmonary fibrosis, interstitial lung abnormalities (ILA), interstitial lung disease, progressive pulmonary fibrosis (PPF), pulmonary fibrosis (PF)

## Abstract

Interstitial lung abnormalities (ILAs) are incidental, non-dependent parenchymal abnormalities detected on chest computed tomography (CT) in individuals without a diagnosis of interstitial lung disease (ILD). Once regarded as non-specific or age-related findings, ILAs are increasingly recognized as clinically relevant entities associated with progression to fibrotic ILD and increased mortality. Emerging molecular and epidemiological evidence indicates that ILAs share key pathogenic pathways with idiopathic pulmonary fibrosis (IPF), the archetypal progressive fibrotic ILD, including alveolar epithelial cell (AEC) stress and injury, innate immune activation, dysregulated repair, fibroblast activation, extracellular matrix (ECM) remodeling, and aging-associated cellular dysfunction. These observations suggest that ILAs may represent the early stage in the continuum of fibrotic lung disease. As such, ILAs provide a valuable opportunity to uncover early disease mechanisms and novel therapeutic targets and enable identification of individuals at increased risk of progression before clinically overt ILD develops. This mini review summarizes emerging mechanistic insights into ILAs and discusses how understanding early disease biology may inform risk stratification and preventative therapeutic strategies.

## Introduction

Interstitial lung abnormalities (ILAs) are incidental, non-dependent parenchymal changes detected on chest computed tomography (CT) in individuals without a prior diagnosis of interstitial lung disease (ILD), involving more than 5% of a lung zone (1, 2). Radiological features include ground-glass opacities, reticulation, traction bronchiectasis, architectural distortion, and non-emphysematous cysts (1–3). The increasing use and improving quality of thoracic CT imaging, particularly within lung cancer screening (LCS) programmes and population health screening cohorts, has led to a substantial rise in the incidental detection of ILAs, with a reported prevalence of 7–10% in the general population and LCS cohorts, increasing to approximately 26% in familial pulmonary fibrosis cohort studies (4).

Although previously considered non-specific or age-related findings, ILAs are now recognized as being clinically significant and are associated with radiological progression and an elevated risk of developing fibrotic lung disease (1, 5, 6). Longitudinal data demonstrate that radiological progression occurs in approximately 20% of individuals over 2 years and 40% over 5 years (1). In addition, long-term follow-up studies have demonstrated progression to a usual interstitial pneumonia (UIP) pattern in approximately 17% of individuals with ILAs over a median period of 11.8 years (6). While in a small UK lung cancer screening study, 40% of individuals with ILAs were subsequently diagnosed with ILD over 5 years (7).

Importantly, ILAs are independently associated with increased all-cause mortality. A recent meta-analysis reported a pooled odds ratio of 3.56 (95% CI 2.19–5.81) over a median follow-up of 5 years (4). Additionally, ILAs have been associated with increased risks of lung cancer, adverse lung cancer outcomes, and cardiovascular disease, even after adjustment for shared exposures such as smoking (8–11). These observations highlight a broader multimorbidity profile and suggest that ILAs may reflect systemic processes linked to aging, inflammation, or environmental injury. However, the persistence of increased mortality after adjustment for major comorbid conditions – including cardiovascular disease, diabetes, chronic kidney disease and malignancy – indicates that these associations do not fully account for the observed risk (12).

A major barrier to effective intervention in fibrotic ILD is delayed diagnosis, as patients frequently present once irreversible extracellular matrix (ECM) deposition and architectural distortion have already occurred (13). ILAs therefore provide an important opportunity to investigate and intervene at the earliest stages of disease. Epidemiological and genetic studies demonstrate that ILAs share key risk factors with idiopathic pulmonary fibrosis (IPF), including increasing age, cigarette smoking exposure, and genetic susceptibility variants such as the MUC5B promoter polymorphism (4, 14). These shared risk factors support the concept that at least a subset of ILAs may represent early or preclinical stages along the biological continuum of fibrotic ILD.

The growing implementation of CT-based lung cancer screening programmes means that ILAs are being detected with increasing frequency (7, 15, 16). Despite this rising prevalence, robust strategies to distinguish individuals with stable ILAs from those at risk of progressive fibrosis remain limited. Improved understanding of the molecular and cellular pathways underlying ILAs may facilitate earlier identification of individuals at highest risk of progression, and inform preventative therapeutic strategies aimed at intercepting fibrotic lung disease before irreversible tissue remodeling occurs. This review discusses the major mechanisms implicated in the development and progression of ILAs, including aging and cellular senescence, genetic susceptibility, epithelial injury and dysregulated repair, immune activation and vascular dysfunction.

## Aging and cellular senescence

Aging is one of the strongest risk factors for both ILAs and fibrotic ILD. The prevalence of ILAs increases markedly with advancing age, even after adjustment for smoking exposure and other environmental risk factors (17, 18). Age-related cellular dysfunction is therefore thought to create a permissive pathological environment in which alveolar epithelial cell (AEC) injury and defective repair responses can initiate lung fibrosis (19).

Telomere dysfunction represents a key molecular hallmark of aging and has been implicated in fibrotic lung disease. Progressive telomere shortening occurs with aging and limits the replicative capacity of somatic cells. Reduced telomere length (20, 21), has been consistently linked with IPF and in asymptomatic individuals at high familial risk for pulmonary fibrosis, indicating that telomere attrition may precede overt clinical disease and function as an inherited susceptibility factor (22–24). Mechanistically, telomere dysfunction is thought to impair the proliferative capacity of type 2 AEC cells following injury, promoting cellular senescence or apoptosis and the release of pro-fibrotic mediators that promote fibroblast activation and ECM deposition (25). Variants in telomere maintenance genes, including TERT, TERC, and RTEL1, are well established in fibrotic lung disease, and carriers may demonstrate early or subclinical interstitial changes on imaging. However, the evidence is more limited in ILAs. Reduced telomere length has been associated with ILAs in population-based studies (26), suggesting that impaired cellular regenerative capacity may contribute to early disease pathogenesis (18, 27), but these associations are observational and do not establish a causal role. Telomere dysfunction should therefore currently be regarded as a plausible susceptibility mechanism rather than an established driver of ILA development or progression.

Further supporting a link between premature aging and ILAs, aging-related biomarkers have been implicated in early disease. Circulating levels of growth differentiation factor-15 (GDF15), a stress-responsive cytokine within the transforming growth factor-β superfamily, increase with age and in response to cellular stressors such as mitochondrial dysfunction and oxidative injury. Elevated GDF15 levels have been associated with both the presence of ILAs and increased mortality in aging population cohorts (18). In addition, GDF15 has been identified as a key mediator of type 2 AEC senescence and is upregulated in both lung tissue and plasma in patients with IPF (28).

## Genetic susceptibility

Beyond aging-related mechanisms, genetic studies provide strong evidence that ILAs share susceptibility pathways with IPF. The strongest common genetic risk factor for IPF is the MUC5B promoter polymorphism (rs35705950), which is also strongly associated with the presence of ILAs, particularly subpleural and fibrotic radiological phenotypes which are at increased risk of progressing to ILD (29–31). This variant leads to increased expression of the gel-forming mucin MUC5B in distal airway epithelial cells. Dysregulated overexpression may impair normal mucociliary transport and epithelial barrier function, potentially promoting persistent epithelial micro-injury following environmental or endogenous insults (30). Carriage of the *MUC5B* risk allele has been shown to increase the risk of ILAs by approximately 2.8-fold and pulmonary fibrosis by 6.3-fold, and has been linked to longitudinal progression of ILAs, particularly in subpleural ILAs and abnormalities evolving toward a UIP pattern (14).

Additional susceptibility loci shared between ILAs, and fibrotic lung disease have also been identified through genome-wide association studies (GWAS). Variants in the genes, *DPP9* (dipeptidyl peptidase-9)*, DSP* (desmoplakin), and *FAM13A* (Family with Sequence Similarity 13 Member A) have demonstrated significant associations with both ILAs and IPF (32, 33). DPP9 encodes an intracellular serine protease involved in immune regulation and inflammasome signaling, DSP is a key structural component of epithelial desmosomes that maintain cell–cell adhesion and epithelial barrier integrity, and FAM13A regulates Rho GTPase signaling pathways involved in cytoskeletal organization and epithelial cell polarity. Taken together, these gene variants indicate dysregulated epithelial repair and epithelial–mesenchymal interactions during tissue remodeling are central to both ILAs and IPF.

In addition to shared susceptibility loci with IPF, GWAS have identified ILA-specific associations that may reflect distinct biological pathways underlying early interstitial remodeling. Variants near importin-11 (IPO11) and FCF1 pseudogene 3 (FCF1P3), a locus linked to ribosome biogenesis and cellular stress responses, have been associated with the presence of ILAs in population cohorts (32). IPO11 is involved in nuclear transport of proteins that regulate cell-cycle control and DNA repair, suggesting a potential role in epithelial stress responses (32). Although the precise biological consequences of these variants remain unclear, their identification suggests that ILAs may arise through multiple genetically driven mechanisms, some overlapping with established IPF pathways, while others may represent distinct molecular pathways of early interstitial remodeling.

Beyond single-variant associations, polygenic risk scores (PRS) derived from IPF susceptibility loci have been associated with both the presence and progression of ILAs in population cohorts. Notably, these associations persist even after exclusion of the MUC5B promoter variant, the strongest genetic risk factor, indicating that multiple additional loci contribute to disease susceptibility. In a large multi-cohort analysis, such a PRS was associated with ILAs (odds ratio ∼1.25) and ILA progression (odds ratio ∼1.16), supporting a shared polygenic architecture between ILAs and fibrotic lung disease (34). Additionally, in asymptomatic first-degree relatives of patients with pulmonary fibrosis, ILAs are detected at substantially higher frequencies than in general population cohorts and are associated with impaired lung function, shorter telomere length, carriage of the MUC5B risk allele, and circulating biomarkers of fibrosis and epithelial stress (22, 35). Longitudinal follow-up also observes that a significant proportion (27%) of these abnormalities progress over time (35). Together, these observations suggest that ILAs arise in lungs that are both biologically aged and genetically susceptible, in which impaired epithelial resilience and defective regenerative capacity amplify the effects of environmental injury and promote maladaptive fibrotic repair responses.

## Epithelial injury and dysregulated repair

Alveolar epithelial cell (AEC) injury is a central initiating event in fibrotic lung disease and circulating protein biomarker studies provide further evidence of epithelial perturbation in ILAs. Large-scale proteomic analyses across multiple population-based and familial cohorts have identified associations between ILAs and epithelial-derived proteins, including surfactant protein B (SFTPB) and surfactant protein D (SFTPD), which reflect AEC injury and barrier dysfunction (36). Additional markers include WAP four-disulfide core domain 2 (WFDC2), CUB domain-containing protein 1 (CDCP1), growth differentiation factor 15 (GDF15), and stratifin (SFN), which are implicated in epithelial stress responses, cell–matrix interactions, and epithelial–mesenchymal signaling (36). Notably, CDCP1 has been linked to transforming growth factor-β (TGF-β) signaling, suggesting that epithelial perturbation in ILAs may already be coupled to early pro-fibrotic pathways (31, 36, 37). WFDC2, GDF15, and CDCP1 have been consistently associated with both the presence and extent of ILAs, reinforcing their relevance to early fibrotic remodeling. Furthermore, increased SFTPB levels were associated with the rs35705950 *MUC5B* promoter polymorphism, suggesting a potential interaction between mucin expression and surfactant homeostasis in distal airways. AEC-associated biomarkers, such as Krebs von den Lungen-6 (KL-6), a mucin-like glycoprotein released by injured or regenerating AECs, have also been associated with ILAs and correlate with imaging abnormalities and lung function, although levels are typically lower than in established ILD (38, 39). These findings support a model in which ILAs represent an early AEC injury state characterized by impaired regenerative capacity, persistent cellular stress, and activation of pro-fibrotic signaling pathways prior to overt architectural distortion. It should be emphasized that although these epithelial markers reflect alveolar epithelial injury and barrier dysfunction, their ability to distinguish progressive from stable ILAs has not been established, and elevated levels may be hypothesized to reflect non-progressive or resolving injury.

Bronchial epithelial gene expression profiling in smokers with ILA provides preliminary evidence that epithelial dysfunction may be established prior to the development of overt fibrosis (40). In this study, airway injury–associated transcriptional signatures were identified, including upregulation of *PPBP* (pro-platelet basic protein), a member of the CXCL7 chemokine family. *CXCL7* has been associated with early wound healing responses and detected in blood and bronchoalveolar lavage samples from patients with IPF (41, 42). It has been hypothesized that this cytokine may contribute to fibrogenesis through recruitment of bone marrow–derived mesenchymal stem cells. Additionally, enrichment of TNF-α signaling pathways in ILAs with a UIP pattern are also observed in IPF, supporting a role for epithelial injury and activation in early disease. TNF-α is a key mediator of epithelial stress responses suggesting that epithelial abnormalities in ILAs may involve both airway and alveolar compartments depending on biological context and exposure history (40, 43).

## Immune activation and early inflammatory signaling

In individuals with ILA, several studies have identified circulating and cellular signatures consistent with low-grade immune activation. Higher peripheral blood monocyte counts have been associated with both the presence and radiological progression of ILAs, supporting a role for monocyte-associated pathways in early disease pathogenesis (44). This observation parallels findings in IPF, where elevated monocyte counts are associated with disease severity and mortality (45). Recruitment of circulating monocytes to the lung is thought to occur via chemokine pathways such as the CCL2–CCR2 axis (46). Monocytes may differentiate into macrophages and contribute to tissue remodeling through pro-fibrotic mediator secretion, although this has been more clearly established in fibrotic lung disease than in ILAs (47).

Circulating levels of inflammatory mediators, including C-reactive protein (CRP), interleukin-6 (IL-6) and tumor necrosis factor receptors (TNFR) have been associated with ILAs or related subclinical interstitial lung injury phenotypes (18, 48). While these markers are not specific to fibrotic lung disease, their presence supports the concept that ILAs arise in the context of persistent epithelial stress and immune activation. In addition, circulating galectin-3, a β-galactoside–binding lectin that promotes fibroblast activation and ECM deposition, has been associated with restrictive lung physiology and ILAs, implicating systemic inflammatory and fibrotic signaling in early disease (49). These mediators are however non-specific and overlap substantially with the systemic low-grade inflammation of aging and multimorbidity, often termed inflammaging (50). No ILA-specific immune signature has yet been defined, and circulating markers alone are unlikely to resolve this ambiguity. Distinguishing immune activation mechanistically linked to early fibrotic remodeling in progressive ILAs from generalized age-related inflammation will require spatially resolved tissue analysis and longitudinal sampling rather than cross-sectional circulating measurements (50).

Studies integrating plasma proteomics with imaging data have further identified enrichment of proteins involved in immune regulation, leukocyte adhesion, and ECM remodeling that are associated with ILAs, including ICAM5 (Intercellular Adhesion Molecule 5) and MMP7 (Metalloproteinase 7) (48, 51). MMP7 is a protease involved in ECM homeostasis, while modulating inflammatory mediators and growth factors. Increased MMP7 levels observed in established fibrotic lung disease support the hypothesis that immune pathways relevant to fibrosis may already be engaged during early, subclinical stages (52).

## Microscopic fibrosis and inflammation in ILAs

Histopathological studies provide important evidence that ILAs are frequently associated with underlying fibrotic change. Analyses of lung tissue obtained from surgical resections or lung explants demonstrate a spectrum of abnormalities, including subpleural fibrosis, fibroblastic foci and honeycombing. Incidental epithelial lesions such as atypical adenomatous hyperplasia have also been reported in some resection-based series but are not considered part of the fibrotic remodeling process (53–55). One study described lymphocytic infiltration as a common feature again suggesting an inflammatory component in early disease (54). Notably, fibrotic changes may be identified even in regions that appear radiologically normal on CT imaging, indicating that structural remodeling can precede overt radiographic abnormalities (53–55). Fibroblastic foci, aggregates of activated fibroblasts and myofibroblasts, are a hallmark of IPF but are not consistently present in all ILAs, suggesting that widespread fibroblast activation is not a universal feature of ILAs (54). Together these findings support a model in which ILAs frequently exhibit early or spatially heterogeneous fibrotic remodeling, with variable degrees of fibroproliferative activity. An important limitation, however, is that these tissue samples are typically derived from selected populations undergoing surgical resection, explant evaluation, or pulmonary nodule assessment, and may therefore not be representative of screen-detected ILAs in the general population.

## Systemic vascular dysfunction

In addition to local lung injury, systemic vascular dysfunction may contribute to the development of ILAs. Epidemiological studies demonstrate strong associations between ILAs and cardiovascular disease, metabolic syndrome, and increased cardiovascular mortality, even after adjustment for smoking and age (11, 12, 15). Histopathological analyses further indicate that vasculopathy is a common feature in ILAs (54), while quantitative CT studies show that pulmonary vascular pruning is associated with both the presence and progression of ILAs, suggesting that early pulmonary vasculopathy may be a feature of preclinical interstitial lung disease (56).

These observations align with findings in IPF, where microvascular loss and vascular remodeling are well recognized and may contribute to disease progression. Together, these data support a model in which vascular dysfunction represents a shared component across the spectrum of fibrotic lung disease, potentially contributing to microvascular injury, tissue hypoxia, and fibroproliferative remodeling. In this context, ILAs may, in some individuals, reflect early manifestations of a broader systemic process involving age-related inflammation and vascular dysfunction, rather than purely localized pulmonary pathology.

## Mechanisms of ILA stability and resolution

Although this review has focused on potential mechanisms driving high-risk, progressive ILA, most ILAs do not progress over conventional follow-up: in a recent serial-CT analysis of a smoking-enriched cohort, 28% showed no radiological progression over approximately 5 years (57), and frank regression, though reported, is uncommon and poorly characterized (58). The biology of non-progression is far less studied than that of progression. Several candidate protective processes can be inferred from IPF and experimental models, but none has been demonstrated in ILAs, and all remain hypotheses for future investigation.

These include competent alveolar regeneration, in which type 2 AECs transit through a Krt8-high intermediate state and complete differentiation to restore the barrier, rather than accumulating as the persistent transitional and KRT5-/KRT17+ aberrant basaloid cells that sustain pro-fibrotic signaling in IPF (59, 60); effective clearance of senescent cells, whose secretory phenotype is fibrogenic and whose removal improves outcomes in experimental fibrosis (61, 62); and intact immune-regulatory networks, although the role of regulatory T cells is contested and they may be either anti- or pro-fibrotic depending on injury stage (63). Defining the determinants of stability, including markers that distinguish self-limiting from progressive injury, is as important as characterizing progression, and is essential if risk stratification is to avoid over-treating the majority whose abnormalities will never progress.

## Discussion

**FIGURE 1:**
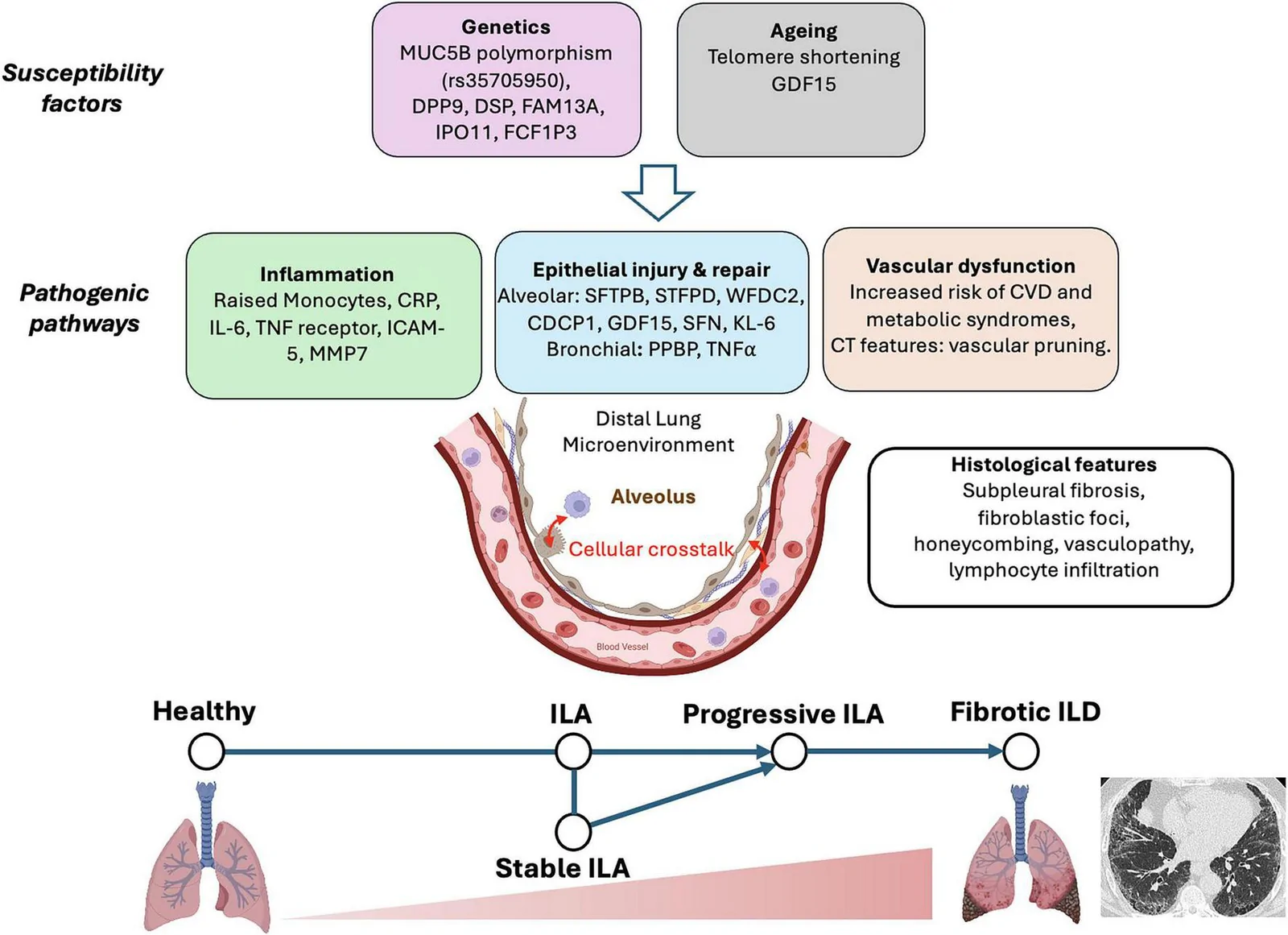
Interstitial lung abnormalities: a mechanistic window into early fibrotic lung disease. CT, computed tomography; CRP, C-reactive protein; IL-6, interleukin-6; TNF receptor, tumor necrosis factor receptors; ICAM-5, intercellular adhesion molecule-5; MMP7, matrix metalloproteinase-7; SFTPB, surfactant protein B; SFTPD, surfactant protein D; WFDC2, WAP four-disulfide core domain 2; CDCP1, CUB domain-containing protein 1; GDF15, growth differentiation factor 15; SFN, stratifin; KL-6, Krebs von den Lungen-6; PPBP, pro-platelet basic protein; TNF-alpha, tumor necrosis factor alpha; MUC5B, mucin 5B; DPP9, dipeptidyl peptidase 9; DSP, desmoplakin; FAM13A, family with sequence similarity 13 member A; IPO11, importin-11; FCF1P3, FCF1 pseudogene 3; CVD; cardiovascular disease.

ILAs are increasingly recognized as a heterogeneous pre-fibrotic state that share key pathogenic mechanisms with established fibrotic ILD, including alveolar epithelial cell stress, impaired regeneration, and activation of pro-fibrotic signaling pathways (Figure 1). A central challenge lies in explaining the marked heterogeneity of ILAs, as only a proportion progress to clinically significant fibrosis. This variability likely reflects differences in genetic susceptibility, aging-related cellular dysfunction, environmental exposures, and the balance between epithelial injury and tissue repair. A recurring limitation is that most biomarkers associated with ILAs have been identified through observational studies and should therefore be interpreted as indicators of biological processes and disease risk rather than established causal mediators of progression.

Recent advances in single-cell and spatial transcriptomics in IPF demonstrate that fibrosis arises within spatially organized multicellular niches composed of interacting epithelial, immune, and mesenchymal cells. Although derived from established disease, this framework is highly relevant to ILAs, which may represent localised microenvironments where these pathogenic interactions are initiated. Defining the cellular composition and signalling networks within these regions may help distinguish ILAs that remain stable from those that progress to fibrotic ILD.

Experimental ex vivo models that preserve tissue architecture, such as human precision-cut lung slices (hPCLS), maintain spatially organized epithelial, immune, and mesenchymal interactions within the native extracellular matrix. Following pro-fibrotic stimulation, hPCLS recapitulate early fibrotic changes - including epithelial injury responses, paracrine signaling, and fibroblast activation - consistent with processes implicated in ILAs, thereby enabling investigation of multicellular networks that precede irreversible tissue remodeling and providing a tractable platform to interrogate potential mechanisms and therapeutic targets relevant to ILAs (64, 65).

From a clinical and translational perspective, the growing recognition that ILAs represent a pre-disease state has important implications. Identifying high-risk phenotypes is therefore essential for developing effective surveillance strategies and enabling early intervention. Radiologic features such as subpleural distribution, reticulation, and traction bronchiectasis are strongly associated with progression, although imaging alone is unlikely to fully capture the underlying biological heterogeneity. Advances in quantitative CT analysis, machine learning, and artificial intelligence offer additional opportunities including deep-learning and texture-analysis approaches to detect subtle radiological features associated with early fibrotic remodeling. Integrating imaging with circulating biomarkers, proteomic signatures, and genetic susceptibility markers may enable development of predictive models capable of distinguishing progressive from non-progressive ILAs and facilitating enrichment of prevention-focused clinical trials.

Overall, ILAs offer a unique window into the earliest stages of fibrotic lung disease. Targeting profibrotic pathways at this stage, prior to irreversible structural remodeling, may provide the greatest opportunity to alter disease trajectory and enable preventative therapeutic strategies in pulmonary fibrosis.
